# Muscle Activation During Grasping With and Without Motor Imagery in Healthy Volunteers and Patients After Stroke or With Parkinson's Disease

**DOI:** 10.3389/fpsyg.2018.00597

**Published:** 2018-04-24

**Authors:** Manuela Kobelt, Brigitte Wirth, Corina Schuster-Amft

**Affiliations:** ^1^Research Department, Reha Rheinfelden, Rheinfelden, Switzerland; ^2^Department of Health Sciences and Technology, ETH Zurich, Zurich, Switzerland; ^3^Interdisciplinary Spinal Research, Department of Chiropractic Medicine, Balgrist University Hospital, Zurich, Switzerland; ^4^Institute of Rehabilitation and Performance Technology, Bern University of Applied Sciences, Burgdorf, Switzerland; ^5^Division of Rehabilitative and Regenerative Medicine, Department of Sport, Exercise and Health, University of Basel, Basel, Switzerland

**Keywords:** stroke, Parkinson's disease, healthy volunteers, motor imagery, muscle activation

## Abstract

**Introduction:** The present study assessed whether motor imagery (MI) produces electromyographic activation in specific muscles of the upper limb during a hand grasping and arm-lifting task in healthy volunteers, patients after stroke, or with Parkinson's disease. Electromyographic (EMG) activation was compared under three conditions: MI, physical execution (PE), and rest. The task is clinically relevant unilateral executed movement using open muscle chains.

**Methods:** In a cross-sectional study EMG activation was measured in four muscles: M. deltoideus pars clavicularis, M. biceps brachii, M. extensor digitorum, M. flexor carpi radialis. MI ability was evaluated with mental rotation, mental chronometry and the Kinaesthetic and Visual Imagery Questionnaire. Cognitive performance was screened with the Mini-Mental State Examination.

**Results:** Twenty-two participants (11 females, age 52.6 ±15.8, age range 21 to 72) were included: ten healthy volunteers, seven patients after stroke (time after stroke onset 16.3 ± 24.8 months), and five patients with Parkinson's disease (disease duration 60.4 ± 24.5 months). Overall Mini-Mental State Examination scores ranged between 27 and 30. An increased EMG activation during MI compared to rest condition was observed in M. deltoideus pars clavicularis and M. biceps brachii across all participants (*p*-value = 0.001, *p* = 0.007). Seven participants (two healthy volunteers, three patients after stroke and two patients with Parkinson's disease) showed a EMG activation during MI of the hand grasping and arm-lifting task in at least one of the target muscles. No correlation between EMG activation during MI and scores of three MI ability assessments were found.

**Conclusions:** The findings suggest that MI can yield subliminal EMG activation. However, that might vary on individual basis. It remains unclear what parameters contribute to or inhibit an EMG activation during MI. Future investigations should determine factors that influence EMG activation, e.g. MI instructions, tasks to imagine, amount of MI training, and longitudinal changes after an MI training period.

## Introduction

Motor imagery (MI) is a key technique in motor learning and motor control facilitating brain plasticity (Shumway-Cook and Woollacott, 2007). MI, which is an established training technique from sports sciences, was recently introduced to the field of neurorehabilitation, in particular to stroke rehabilitation (Braun et al., 2013; Guerra et al., 2017). Decety and Grezes defined MI as “…a dynamic state during which the representation of a given motor act is internally rehearsed without any motor output.” (Decety and Grezes, 1999).

From research with healthy volunteers and professional athletes we know that MI can improve physical performance and learning (Mulder et al., 2004). MI uses two approaches to positively influence physical performance: (1) optimising psychological processes on the regulation of autonomous excitation, reduction of anxiety, strengthening self-efficacy and self-confidence, and motivation in general (Zentgraf and Munzert, 2014); (2) modulating the inhibition or excitability in different body systems, e.g., brain, muscles, autonomic nervous system. Recently, Bajaj et al. found a significantly increased regional connectivity between the premotor cortex and the primary motor cortex after a 3-week MI intervention in stroke patients (Bajaj et al., 2015).

Reviews from the field of neurorehabilitation highlighted the potential neural correlates and the effect of specific brain lesions on MI ability and therefore its consequences in the rehabilitation of patients after stroke or with Parkinson's disease (PD) (McInnes et al., 2016; Caligiore et al., 2017; Tong et al., 2017). Tong et al. summarised the potential effect of MI for upper and lower limb training for stroke patients. However, McInnes et al. found that patients with a brain lesion in the parietal and frontal lobes might not benefit from MI training. However, just recently, Guerra et al. summarised the beneficial effect of MI on activities of daily living, balance, lower limbs and gait, as well as upper limb function without limitation to specific brain lesions (Guerra et al., 2017). For patients with PD, Caligiore et al. postulated that the effect of MI in PD could be influenced by the duration of the disease. Heremans et al. showed that MI ability was preserved in patients with PD (Heremans et al., 2011a). Moreover, the authors demonstrated that MI ability was improved through visual cues (Heremans et al., 2011b). Furthermore, MI might positively influence cognitive and motor function performance in patients with PD if combined with physical execution (PE) in group setting sessions (Tamir et al., 2007).

Different theories try to explain why muscle activity signals might be detectable during MI (Guillot et al., 2010). Guillot et al. concluded that it remains unclear if an increase in EMG activity level is associated with motor performance improvement, level of MI expertise, MI vividness, selected MI mode and perspective (Guillot et al., 2010). Dickstein et al. investigated EMG activation during MI in a lower limb movement task in six patients after stroke and nine healthy volunteers (Dickstein et al., 2005). In six out of 15 participants (three participants per group) they found an EMG activation during MI and PE in the medial gastrocnemius muscle. Two recent studies showed the importance of a neuromuscular coupling of MI. Mateo et al. trained six tetraplegic spinal cord injured patients for 45 min three times a week for 5 weeks with MI to improve the “tenodesis grasp” (Mateo et al., 2015). After the training period the wrist extension angle increased and the activated voxels in the contralateral sensorimotor cortex were similar to the six healthy controls. Furthermore, Page et al. evaluated the feasibility of a functional electric stimulation device (Mentamove, Karlsfeld, Germany) in a stable chronic stroke population (Page et al., 2015). The device used MI-generated muscle activation from paretic muscles to initiate functional electric stimulation of these muscles. This technique may become a valuable rehabilitation tool, however, as this has only, to our knowledge, been investigated in a stable stroke population it would be important to also explore whether EMG can be elicited during MI in other patient populations as well, e.g., with PD.

Therefore, in the present study, we aimed to investigate whether EMG activation could be detected during MI of an upper limb task in a chronic stroke population (Dickstein et al., 2005; previously shown by Page et al., 2015) but also in patients with PD, and to compare them with a healthy group.

The movement investigated by Dickstein et al. was an alternating, bilaterally executed, lower limb task involving closed muscle chains and only few degrees of freedom. In contrast, we were interested in a hand grasping and arm-lifting task, which is a clinically relevant, unilateral executed movement task using open muscle chains. Therefore, the main goal of the present study was to explore the muscle activation of an upper limb task in patients after stroke or with PD during three conditions: MI, PE, and rest. Further, the findings were compared to the EMG activation in healthy volunteers. We hypothesised that an EMG activation during MI and PE of the hand grasping and arm-lifting task would be detectable. Furthermore, based on the findings from Dickstein et al. we expected that EMG activation during MI would differ from the rest condition for all healthy volunteers, patients after stroke, and patients with PD due to their subcortical lesion. All participants underwent a battery of MI ability assessments including mental rotation, mental chronometry, and the Kinaesthetic and Visual Imagery Questionnaire. As a subgoal, we analysed personal data and MI ability assessments of participants, which showed EMG activation during MI.

## Materials and methods

### Study design

The study comprised a cross-sectional investigation with two measurement sessions. On the first measurement session participants underwent a cognitive, handedness, and MI ability screening. Additionally, participants were screened regarding their ability to perform a hand grasping and arm-lifting task, and they were introduced to MI practice. On the second measurement session EMG activation was recorded during the hand grasping and arm-lifting task under two conditions: MI and PE. The study was conducted in accordance with the Declaration of Helsinki and was approved by the ethics committee of the Canton Aargovia, Switzerland (Ref. Nr. EK: 2013/034).

### Selection criteria and participant recruitment

Selection criteria for all three participant groups are presented in Table 1. Patients were recruited via the rehabilitation clinic's database and treating physiotherapists. Recruitment comprised the following steps: (1) Patient entry lists for inpatients and patients attending the neurological day care centre were screened on a regular basis by the authors. Potential participants were identified and their clinical reports were screened. Treating physiotherapists were consulted regarding the patients' clinical symptoms. Healthy participants were recruited via leaflets. (2) Participants were informed about the on-going study in oral and written form. (3) After providing written informed consent participants were invited to two measurement sessions. Healthy volunteers were recruited with information leaflets provided within the clinic and the medical fitness centre. All healthy volunteers and patients participated voluntarily and gave written informed consent before data collection began.

**Table 1:** Participant selection criteria.

**Participant**	**Inclusion criteria**	**Exclusion criteria for all groups**
All participants	- Males and females older than 18 years - Able to sit independently with closed eyes on a normal chair - Able to perform the hand grasping and arm lifting task without external help - Provide written informed consent	- Additional neurological, psychological, or psychiatric disease, severe pulmonary and cardiovascular diseases - Severe pain - Severe deformation of joints of the upper limb with arthritic origin - Present impairments in cognition and communication
Healthy participants	- No neurological or psychological disease	
Patients after stroke	- Patients in the subacute or chronic phase after first-ever stroke - Present an arm and hand paresis	
Patients with Parkinson's disease (PD)	- Patients with an idiopathic PD - No treatment with deep brain stimulation	

### First measurement session: assessments and MI introduction

**Figure 1:**
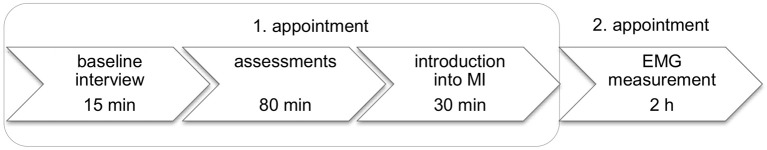
Measurement sessions and study procedure. EMG: electromyography; h: hour; MI: motor imagery; min: minute.

Data were collected in the rehabilitation centre Reha Rheinfelden in Switzerland between August and October 2013. Participants were tested individually (Figure 1). Besides the personal, medical, and MI experience the following data were collected:

To assess cognitive function, the **Mini-Mental State Examination** (MMSE) was conducted in a face to face meeting with each participant (Tombaugh and McIntyre, 1992). Based on our previous experience, 20 points or more had to be achieved for study inclusion (Schuster et al., 2012).

To assess hand laterality, the **Edinburgh Handedness Inventory** was used (Oldfield, 1971). The questionnaire included 12 daily activities were participants had to determine their preferred hand (right/left).

The **Hoehn and Yahr scale** was applied to all patients with PD to classify the disease state (Hoehn and Yahr, 1967).

#### Hand grasping and arm lifting task

Participants had to perform a hand grasping and arm-lifting task with the dominant hand in healthy volunteers and with the more affected hand in patients after stroke and with PD. We were interested if we could detect muscle activation in the paretic limb during MI (Dickstein et al., 2005). The movement was executed as follows: (1) participants were sitting in front of a table with the back leaned against the backrest of a chair without armrests, (2) both forearms rested in 90 degree angle on the table with hands open, (3) an empty plastic cup was positioned centrally in front of the participants with a distance of 0.3 m to the table edge, (4) one hand grasped the cup, moved it to the lips and placed it back on the table, (5) after each execution arm and hand moved back to the starting position. The task was also used to evaluate participants' mental chronometry as described below.

To evaluate participants ability to create a mental image and therefore to be included in the study, participants **motor imagery ability** was evaluated with three assessments: **mental rotation** (Moseley, 2004), **the Kinaesthetic and Visual Imagery Questionnaire** (Malouin et al., 2007), and **mental chronometry** (Malouin et al., 2008). Two out of the three MI ability assessments had to be scored satisfactorily leading to the inclusion of the participant into the study.

#### Mental rotation (MR)

Pictures of hands and feet (Moseley, 2004) (recognise flash card, neuro orthopaedic institute, Adelaide City West, Australia) were presented in four different perspectives (palm, back of the hand, ulnar and radial side), two per body side (left, right) were shown in four various rotations (0, 90, 180, 270 degrees). In total, 64 pictures of hands and feet were presented on a computer screen in a randomised order for 10 s each. Of all pictures, 75% of the pictures had to be recognised for a satisfactory score, as recommended by Sharma et al. (Sharma et al., 2008).

#### The kinaesthetic and visual imagery questionnaire (KVIQ)

The KVIQ was developed for patients with sensorimotor impairments. The KVIQ is a valid and reliable questionnaire that can be applied in healthy volunteers, patients after stroke, or with PD (Malouin et al., 2007; Randhawa et al., 2010). The questionnaire is available as a short version (KVIQ-10), which composes 10 items, five per scale. The scales are defined as both visual and a kinaesthetic 5-point Likert scales ranging from 1 to 5 (1 = “no image”/“no sensation”, 5 = “image as clear as seeing it”/“as intense as making the movement”). A scoring of 30 out of 50 was deemed a satisfactorily threshold.

#### Mental chronometry (MC)

MC is a reliable method to examine the temporal structure of MI in healthy volunteers and post stroke patients (Malouin et al., 2008). We used the study task ‘Hand grasping and arm lifting task' to evaluate participants’ MC. The examiner demonstrated the task once to each participant. Subsequently, participants had the opportunity to train the ‘Hand grasping and arm lifting physically and mentally. For the MC evaluation, time needed to perform the task was recorded during three task blocks for each condition (PE, MI) starting with PE. Before starting and finishing the MI condition, participants knocked once with the non-involved hand as a sign for starting/stopping time measurement. A ratio of 1 ± 0.5 was deemed a satisfactorily threshold. Each block in the MI condition was rated for vividness and sensation on the 5-point KVIQ-10 subscales (Di Rienzo et al., 2014).

#### Introduction to MI

At the end of the first measurement participants were introduced theoretically and practically to the concept of MI. A 30 min session was given based on the MI introduction programme by Wondrusch and Schuster-Amft (2013). The examiner gave an overview on the theoretical aspects and practical MI exercises were performed. Additionally, all participants received an exercise sheet describing the MI performance of the hand grasping and arm-lifting task. To familiarise themselves with the MI technique and the performance of the hand grasping and arm-lifting task, participants were recommended to practice the task two to three times per day until the second appointment 2–7 days later. The frequency of practice was neither recorded nor analysed.

### Second measurement session: electromyographic assessment and processing

#### Preparation

Participants' skin preparation and electrode placement were based on the Surface Electromyography for the Non-Invasive Assessment of Muscles (SENIAM) recommendations (Hermens et al., 2000). Electrodes were placed in an inter-electrode distance of 2 cm on the four involved upper limb muscles: M. deltoideus pars clavicularis, M. biceps brachii, M. extensor digitorum, and M. flexor carpi radialis. Two bipolar electrodes were placed parallel to the muscle fibres (Bischoff et al., 2008).

#### EMG measurement

EMG signals were recorded using the wireless device Myon320 (Prophysics AG, Zurich, Switzerland). Data were collected with a sampling frequency of 3 kHz and pre-amplified by a factor of 1,000. During the recordings, signals were displayed using LabView (Service Package 1, 2011, National Instruments, Austin, USA). The experimental protocol was adopted from Dickstein et al. (2005): Three task blocks each comprising three task trials were recorded for PE and MI conditions alternatingly, starting with PE. Between recording blocks a pause of at least 30 s was made. Furthermore, the trial pace of the tasks for both MI and PE were determined by a metronome (Dickstein et al., 2005). In the present study, the metronome speed was set to the individual's average execution speed when performing the task physically plus 20%. During the preparation phase of the present study, almost all volunteers selected the +20% among the randomly added +10%, +20%, or +25% time needed to physically perform the upper limb task as the most convenient metronome rhythm for the mental execution of the task.

**Figure 2:**
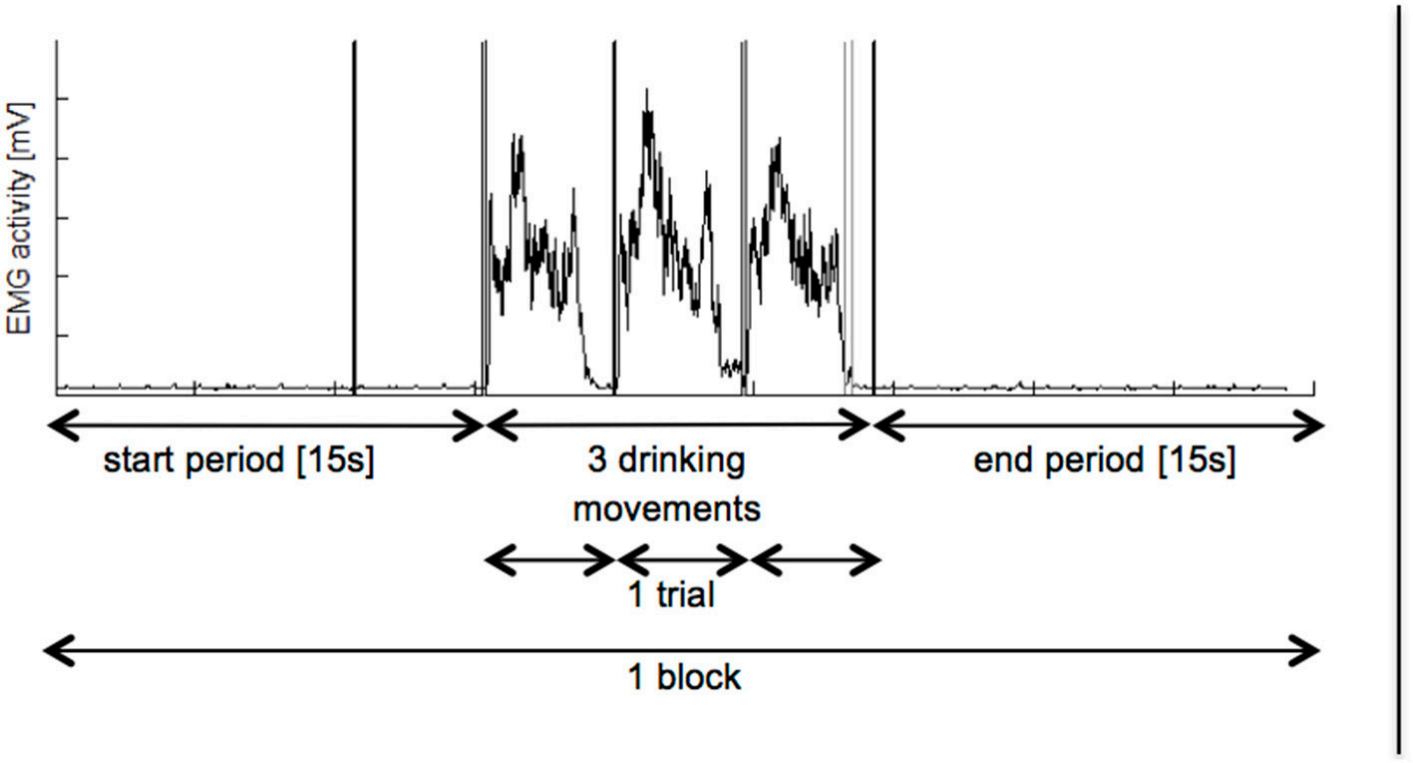
Overview on recorded EMG activation. Each participant had to perform 3 blocks of PE followed by 3 blocks of MI. Whereas, one block consist of a 15 s start period, three hand grasping and arm lifting movements and a 15 s end period. Black vertical lines represent metronome beats and grey line displays manual trigger. EMGm electromyography; mV, millivolt, s: seconds.

1. **Physical Execution**. Before the EMG recording started, the examiner demonstrated the hand grasping and arm lifting task and participants practiced the task once. Before and after each task block, participants were asked to place their hands and arms relaxed in the starting position for 15 seconds to record the rest condition. The rest condition was marked in the data with a recording system trigger.
2. **Mental Execution**. The examiner presented standardised and detailed MI instructions (available from the corresponding author) live during data recording. During MI, participants could keep their eyes closed and were encouraged to use the internal perspective as well as the kinaesthetic modality. All participants received the following instructions: ‘…*, feel yourself grasping the glass and lifting it to your mouth, let the cup touch your lips and bring it back to the table. Then feel how your hand/arm are placed back in the starting position. Try to imagine the same after the second and third metronome beat. Try to feel yourself executing the movements, but do not make any actual movements, just feel yourself lifting the glass.’* For the duration of each MI block, the examiner observed the participants' upper limb to check for potential in arm, hand, or finger movements as suggested by Alkadhi et al. (2005). Finally, to control for the correct MI performance participants were asked to describe their imagination and to grade MI quality after each task block based on the visual and a kinaesthetic 5-point Likert scales of the KVIQ-10 ranging from 1 to 5.
3. **Data Processing**. For each participant, six four-channel EMG data files, three for each condition (PE and MI) were recorded. The metronome beats defined the three consecutive movement trials and a manual trigger labelled the last period in each file (Figure 2). In a second step, the EMG signals were analysed in MATLAB (R2012b, MathWorks, Natick, USA, RRID:SCR_001622). To reduce motion artefacts a high pass filter was applied (10 Hz) (Clancy et al., 2002). To determine the EMG amplitude the signal was rectified and smoothed with a moving average of 100 ms (Konrad, 2005).

#### Sample size and statistical analyses

Our sample size was based on the investigation of Dickstein et al. The authors included nine healthy volunteers and six patients after stroke (Dickstein et al., 2005). We targeted a similar sample size for our three groups: healthy volunteers, patients after stroke, and patients with PD.

### Main goal analysis

#### EMG activation during MI and PE

Each participants' average EMG activation was determined in the three task trials of one block for all four muscles. Each recording was time normalised to 1,000 points. Average EMG activation during MI, PE, and rest was calculated for each muscle and each participant. The Wilcoxon signed rank test was applied as *post-hoc* analysis to the results of the Friedman's analysis of variance (non-normal distribution, related samples) including a Bonferroni correction for three comparisons (*p* = 0.0167) (Friedman, 1937; McLaughlin and Sainani, 2014). In one stroke patient only two out of three task blocks with the MI condition could be recorded and therefore, six instead of nine trials were analysed.

#### Detailed EMG activation analysis during MI

Each trial was analysed individually:

- the MI amplitude average in each task block was compared to the average amplitude of the corresponding rest condition;
- activation during MI was verified by comparing the processed EMG signal against a threshold of 1.5 times of the standard deviation (SD) of the processed EMG signal during the rest condition.

The EMG activation threshold of 1.5 × SD was selected based on Hodges and Bui (1996), who compared different computer-based methods including thresholds of 1, 2, 3 times of the rest EMG standard deviation as EMG onset marker. The authors suggested to use visual inspection of EMG signals to determine the appropriate muscle activation onset threshold. Following their recommendation, we visually identified EMG activation patterns to verify the threshold setting. Visually identified EMG activation pattern were verified by a comparison with the calculated threshold of 1.5 times of the SD of the rest condition.

### Subgoal analysis

#### Results of MI ability assessments

The results of each assessment (MR, MC, KVIQ) were processed individually. For MR, the number of correctly identified hand and feet flash cards was counted. For MC, the ratio (MI/PE) of the time needed to imagine and physically execute the task was calculated. For the KVIQ-10, the average scores of the visual and kinaesthetic subscales were calculated.

#### Self-rating MI quality

The average values over all trials of the visual and kinaesthetic subscales were analysed and are presented as mean and standard deviation.

Statistical analyses were performed using MATLAB (R2012b, MathWorks, Natick, USA, RRID:SCR_001622) and SPSS (Statistical Package for the Social Sciences, version 21, IBM, Armonk, New York, USA, RRID:SCR_002865). Descriptive statistics were calculated for participants' characteristics and assessment scores. Normal distribution was verified using the Kolmogorov-Smirnov test. Correlations among MI ability assessments, self-rating MI quality, and EMG activation during MI was calculated with the Spearman's rank correlation coefficient. Significance level was set to *p* ≤ 0.05.

## Results

In total, 22 out of 24 participants (11 females and 11 males) completed both measurement sessions and were included in the data analyses. Two PD patients had to be excluded because they did not meet the previously described inclusion criteria of the MI ability assessments. One of the two excluded PD patients did not achieve 30 points in the KVIQ-10 assessment and achieved a ratio higher than 1.5 in the mental chronometry assessment. The second PD patient only recognised 55% of the hand and feet pictures and was not able to perform the KVIQ-10. Included participants comprised 10 healthy subjects, seven patients after the first-ever stroke and five patients with PD (Tables 2, 3A,B). An overview on the location of the brain lesion in patients after stroke is provided in Table 4. Patients and healthy volunteers were not age-matched.

**Table 2:** Group characteristics.

	**Parkinson's disease (*n* = 5)**	**Stroke (*n* = 7)**	**Healthy volunteers (*n* = 10)**	**Total (*n* = 22)**	**H(2)**	***p*-value**
Age	65.4 ± 6.0 67, 55–70	53.7 ± 16.3 56, 24–72	45.4 ± 15.4 51, 21–62	52.6 ± 15.8	6.54	**0.038**
Time of disease [M]	60.4 ± 24.5 56, 31–91	16.3 ± 24.8 6, 2–70	n.a.	n.a.	n.a.	n.a.
MMSE	28.6 ± 1.1 29, 27–30	28.7 ± 0.8 29, 28–30	29.2 ± 0.8 29, 28–30	28.9 ± 0.9	1.95	0.378
MI self-rating (max. 5)	3.0 ± 1.0 3.0, 2.0–4.3	3.3 ± 0.7 3.5, 2.0–4.0	3.3 ± 0.6 3.1, 2.5–4.2	3.2 ± 0.7	0.53	0.766
MI practice trials	7.2 ± 4.8 7, 2–15	6.4 ± 4.9 7, 0–13	6.2 ± 1.5 6.5, 4–8	6.5 ± 3.5	0.18	0.914
Mental rotation (0-64)	61.0 ± 1.0 61, 60–62	57.1 ± 4.7 57, 51–64	63.2 ± 1.1 64, 61–64	60.8 ± 3.8	11.07	**0.004**
Mental chronometry PE [s]	4.9 ± 2.3 4.3, 3.5–8.9	3.9 ± 0.8 3.5, 3–5.5	3.5 ± 0.7 3.5, 2.4–4.5	4.0 ± 1.3	3.45	0.178
Mental chronometry MI [s]	5.4 ± 4.5 3.9, 2.8–13.4	3.9 ± 0.8 4.0, 2.8–5.2	3.3 ± 0.9 3.1, 2.1–5.0	4.0 ± 2.2	1.8	0.406
Ratio Mental chronometry ($\frac{MI\;time}{PE\;time}$)	1.1 ± 0.28 0.9, 0.79–1.5	1.03 ± 0.28 1.13, 0.92–0.95	0.97 ± 0.30 0.94, 0.81-1.07	0.99 ± 0.28	0.19	0.910
KVIQ visual (5-25)	17.4 ± 2.9 19, 13–20	18.0 ± 5.2 17, 11–24	18.5 ± 4.3 19, 12–24	18.1 ± 4.1	0.32	0.852
KVIQ kinaesthetic (5–25)	16.4 ± 2.5 15, 14–20	14.4 ± 5.0 15, 6–20	14.2 ± 3.9 14, 9–21	14.8 ± 4.0	1.21	0.545
KVIQ kinaesthetic + visual (10–50)	33.8 ± 4.8 33, 28-40	32.4 ± 7.8 34, 19-41	32.7 ± 4.7 31, 27-40	32.9 (±5.6)	0.32	0.854

*Numbers indicate mean, standard deviation, median, and range. P-values in bold represent significant differences among groups. H: Kruskal-Wallis test value with degrees of freedom in brackets; kin: kinaesthetic; KVIQ: Kinaesthetic and Visual Imagery Questionnaire; M: time of disease in months; MChr: mental chronometry; MI: motor imagery; MI quality self-rating (average value of visual and kinaesthetic self-rating after each trial during MI); MMSE: Mini-Mental State examination; MRot: mental rotation; practice MI: mental practice of hand grasping and arm lifting task at home; n.a.: not applicable; PE: physical execution; s: seconds; vis: visual. P-values of age and mental rotation indicate a significant difference between groups: Age: difference between healthy volunteers and patients with PD, mental rotation: difference between healthy volunteers and patients after stroke or with PD*.

**Table 3:** **(A)** Detailed characteristics of each participant without EMG activation during MI. **(B)** Detailed characteristics of each participant with EMG activation during MI.

**Diagnosis**		**Age category**	**Time disease [M]**	**Affec. side**	**H&Y**	**Education**	**Occupation**	**Handedness**	**MMSE**	**MRot**	**MChr E [s]**	**MChr MI [s]**	**Ratio MChr [MI/PE]**	**KVIQ vis**	**KVIQ kin**	**KVIQ kin+vis**	**Vis (SR)**	**Kin(SR)**
**Max. value**									**(0–30)**	**(0–64)**				**(5–25)**	**(5–25)**	**(10–50)**	**(1–5)**	**(1–5)**
**(A)**																		
	3	7	31	R	1	5	3	L	27	60	4.27	3.02	0.71	16	15	31	3.0	1.3
	3	10	91	L	1	1	4	L	28	62	3.51	2.77	0.79	19	18	37	4.0	4.7
	3	10	56	R	1	5	5	R	29	62	4.32	3.88	0.90	20	20	40	3.7	3.7
	2	6	2	L	n.a.	1	4	R	29	53	3.49	5.21	1.49	11	14	25	3.0	2.7
	2	1	3	R	n.a.	3	2	R	28	57	4.17	3.13	0.75	13	6	19	2.0	2.0
	2	8	7	R	n.a.	1	6	R	30	54	3.02	3.95	1.31	21	20	41	3.3	4.0
	2	9	23	L	n.a.	1	4	R	29	60	3.37	3.61	1.07	17	17	34	3.7	2.7
	1	8	n.a.	n.a.	n.a.	5	4	R	30	62	2.69	2.61	0.97	17	13	30	3.0	2.0
	1	1	n.a.	n.a.	n.a.	2	4	R	30	64	3.61	3.78	1.05	18	21	39	4.0	4.3
	1	9	n.a.	n.a.	n.a.	4	5	R	29	63	3.43	1.97	0.57	24	16	40	4.0	4.0
	1	8	n.a.	n.a.	n.a.	4	4	R	28	61	4.21	3.97	0.94	24	9	33	5.0	2.3
	1	2	n.a.	n.a.	n.a.	2	5	R	29	64	4.18	4.09	0.98	13	14	27	3.7	2.7
	1	7	n.a.	n.a.	n.a.	2	6	R	29	64	2.92	2.95	1.01	15	14	29	3.3	2.7
**(B)**																		
^*^	3	10	79	L	1.5	2	1	R	30	61	3.69	4.02	1.09	19	14	33	3.0	3.0
^*^	3	10	45	L	1	2	1	R	29	60	8.93	13.39	1.50	13	15	28	2.3	1.3
^*^	2	10	70	L	n.a.	1	6	R	29	61	5.46	4.28	0.78	16	19	35	4.0	3.3
^*^	2	5	3	L	n.a.	1	5	R	28	64	3.36	2.79	0.83	24	10	34	3.3	3.0
^*^	2	11	6	R	n.a.	2	1	L	28	51	4.08	4.06	1.00	24	15	39	4.0	4.0
^*^	1	5	n.a.	n.a.	n.a.	2	1	L	30	64	2.45	3.71	1.51	21	17	38	4.0	3.7
^*^	1	8	n.a.	n.a.	n.a.	4	4	R	28	64	3.44	4.80	1.40	20	9	29	2.7	3.0
^†^	1	1	n.a.	n.a.	n.a.	1	5	R	29	64	4.50	2.87	0.64	21	11	32	4.0	3.7
^†^	1	6	n.a.	n.a.	n.a.	3	5	R	30	62	3.82	2.67	0.70	12	18	30	2.0	4.0

Age category: 1 = 20–25, 2 = 26–30, 3 = 31–35, 4 = 36–40, 5 = 41–45, 6 = 46–50, 7 = 51–55, 8 = 56–60, 9 = 61–65, 10 = 66–70, 11 = 71–75. Affected side: 1: right, 2: left; Diagnosis: 1: healthy, 2: stroke patient, 3: patient with Parkinson's disease; Education: 1: apprenticeship, 2: professional college, 3: university, 4: diploma, 5: grammar and middle school; PE, physical execution; G (gender), M: male, F: female; Handedness: R: right, L: left; H&Y (Hoehn and Yahr-scale): 1: Unilateral symptoms only, 1.5: Unilateral and axial involvement; kin: kinaesthetic; KVIQ: kinaesthetic and visual imagery questionnaire; M: time of disease in months; MChr: mental chronometry; MI: motor imagery; MMSE: mini-mental state examination; MRot: mental rotation; occupation: 1: leader in the private sector, 2: scientist, 3: technicians and associate professionals, 4: office worker, 5: service occupation, 6: craftsman; n.a., not applicable; s, seconds; vis, visual; vis(SR) and kin(SR), average value of self-rating after each trial during MI on visual and kinaesthetic subscales;

* *Participants with electromyographic activation during MI above the threshold value, defined as multiple (1.5) of the standard deviation of the normed rest condition*.

† *Participants with EMG activation during MI below the threshold. Numbers in brackets indicate range of possible scores*.

**Table 4:** Location of brain lesions for the seven included patients after stroke.

	**ID**	**Location**	**Type**
	6	Corona radiate, right	hemorrhagic
^*^	7	Basal ganglia	ischemic
	8	Thalamus and tectum mesencephali	hemorrhagic
^*^	9	Area arteria cerebri media and left arteria cerebri anterior	hemorrhagic
^*^	10	Frontal cortex, left	hemorrhagic
^*^	11	Area of arteria cerebri media, left	hemorrhagic
	12	Arteria cerebri media, right	hemorrhagic

* *Participants with scores <60 in mental rotation. ID: patient identification number*.

### Main goal analysis

#### EMG activation during MI and PE

**Figure 3:**
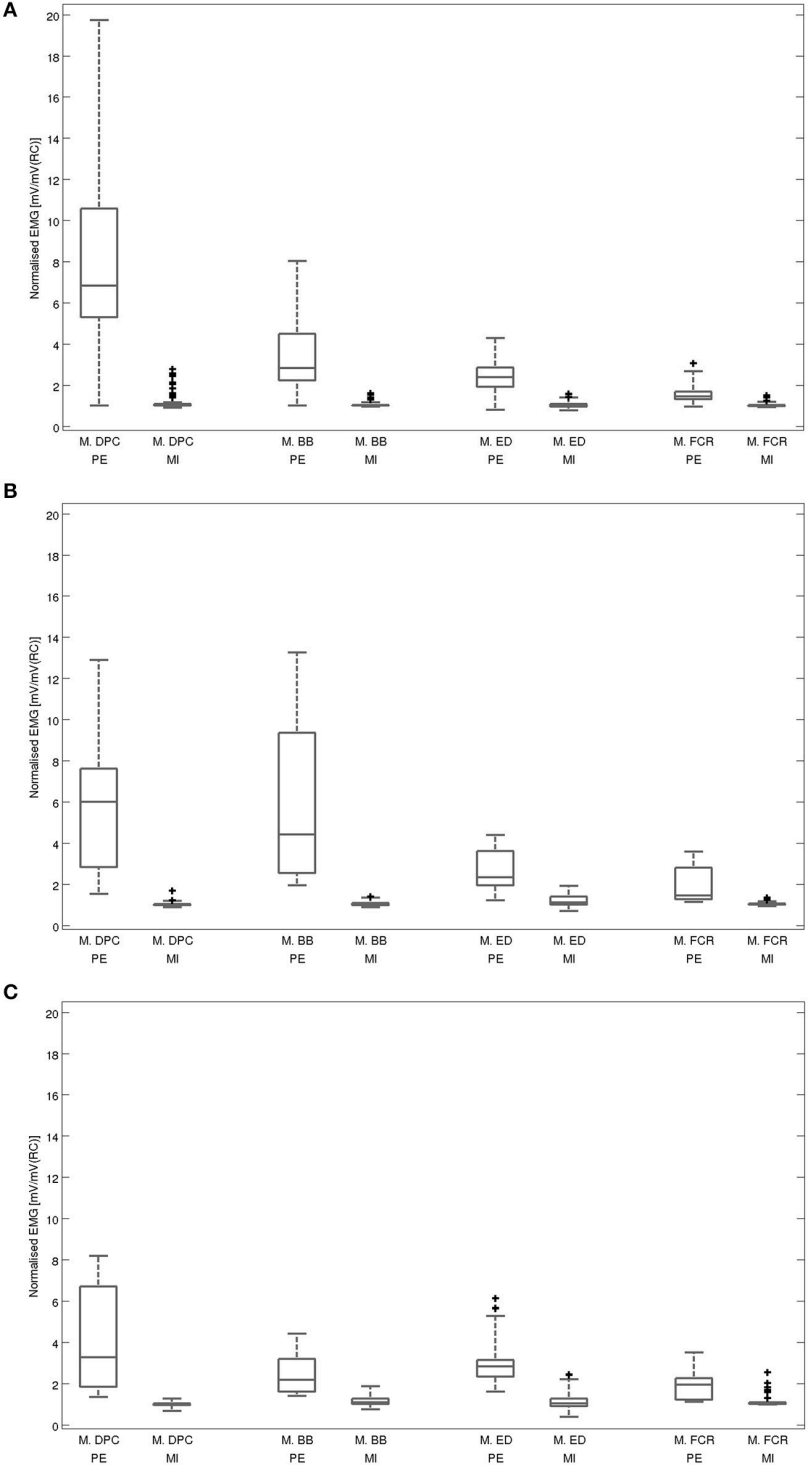
Averaged EMG activation during physical execution and MI within each muscle: **(A)** for all healthy volunteers, **(B)** for all patients after stroke, **(C)** for all patients with Parkinson's disease. Muscles: M. DPC: M. deltoideus pars clavicularis, M.BB: M. biceps brachii, M.ED: M. extensor digitorum, M.FCR: M. flexor carpi radialis; PE: execution, MI: motor imagery, mV: millivolt, RC: rest condition. Illustrated outliers are larger or smaller than 1.5x interquartile range.

**Figure 4:**
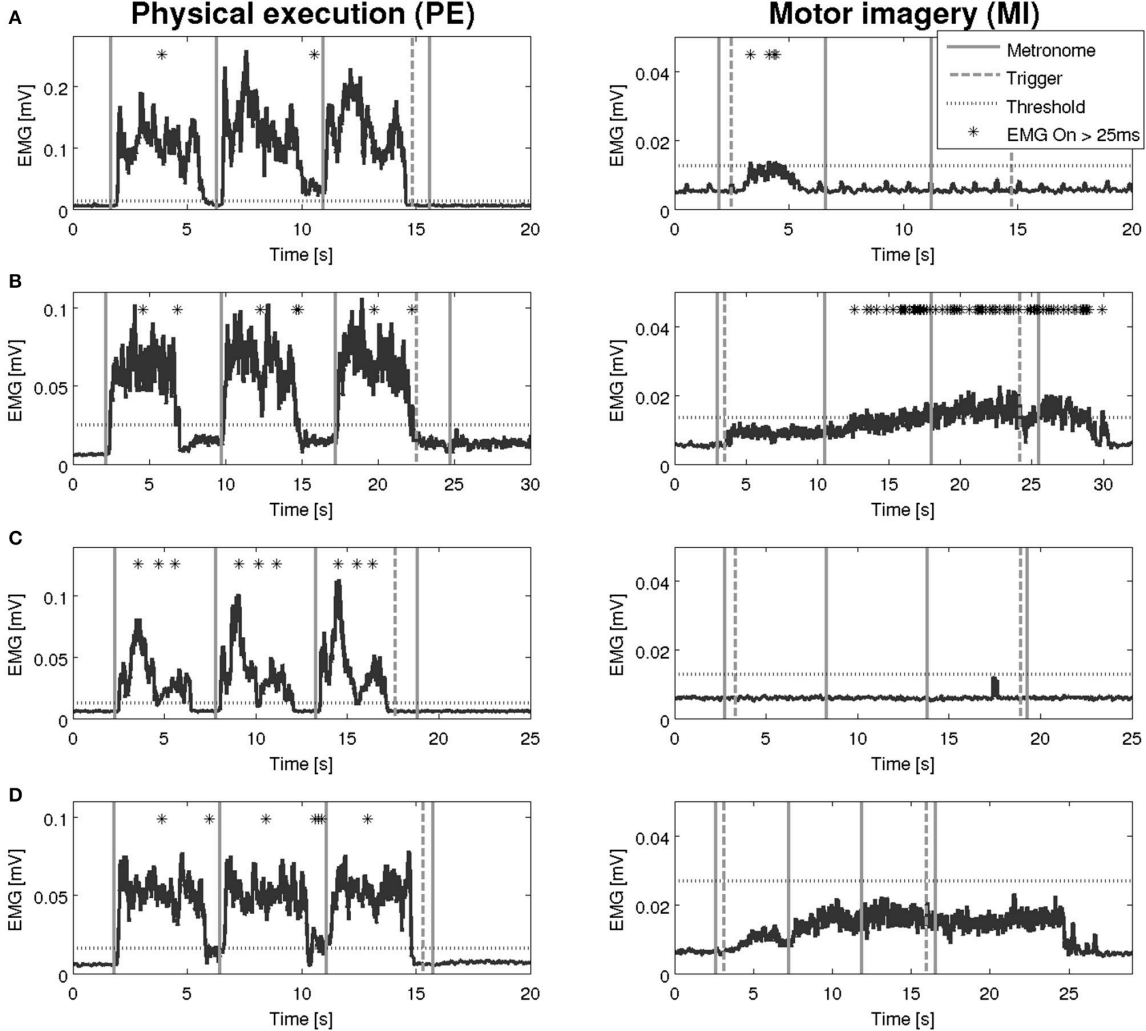
EMG activation during physical execution and MI of four healthy volunteers. **(A)** Healthy volunteer, M. deltoideus pars clavicularis with an EMG activation above the threshold. **(B)** Healthy volunteer, M. flexor carpi radialis with no EMG activation above the threshold. **(C)** Healthy volunteer, M. deltoideus pars clavicularis with an EMG activation above the threshold. **(D)** Healthy volunteer, M. deltoideus pars clavicularis with no EMG activation above the threshold.

**Figure 5:**
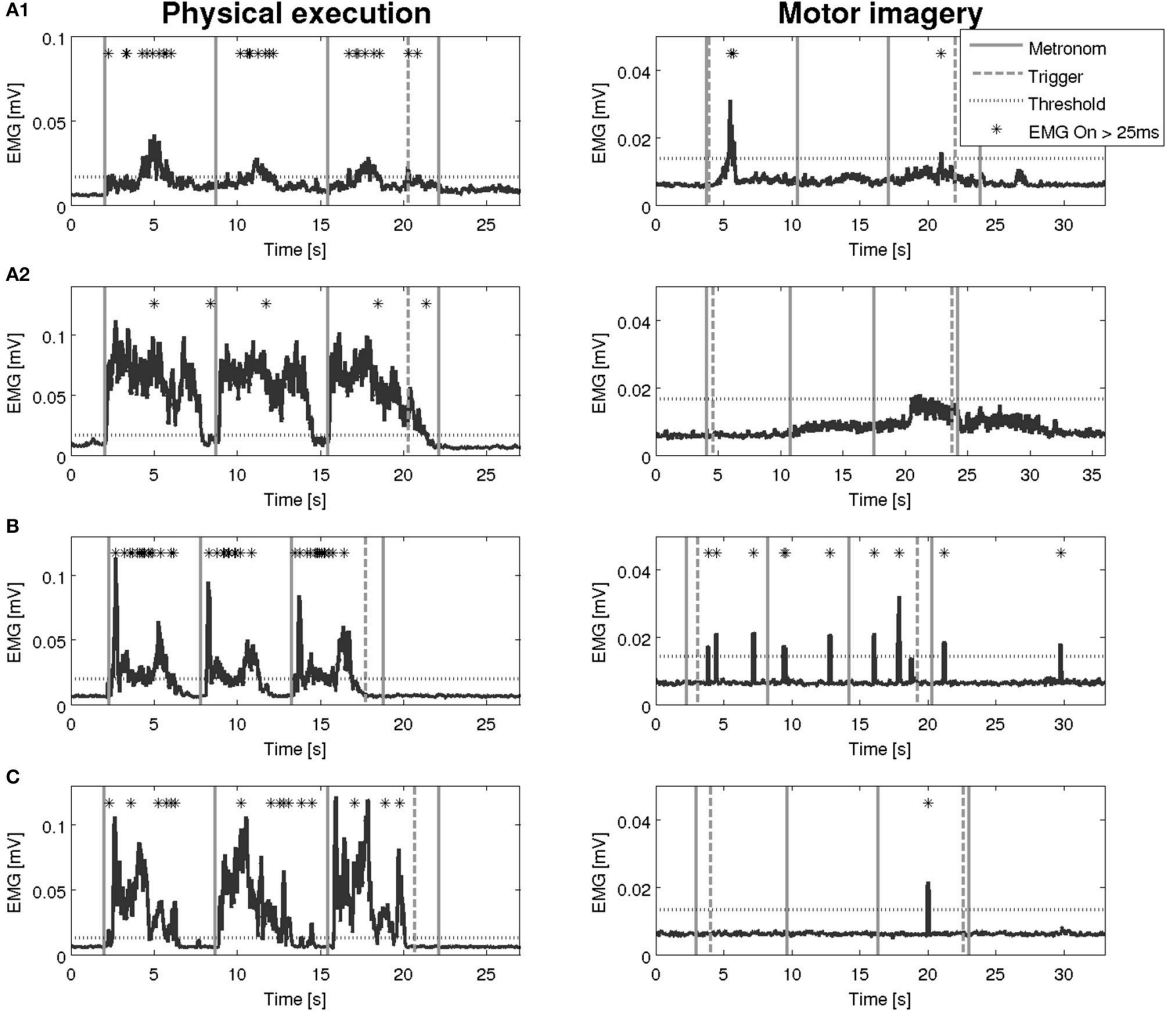
EMG activation during physical execution and MI of three patients after stroke. **(A1,A2)** Patient after stroke, M. extensor digitorum with an EMG activation above the threshold, M. deltoideus pars clavicularis with no EMG activation above the threshold. **(B)** Patient after stroke, M. flexor carpi radialis with an EMG activation above the threshold. **(C)** Patient after stroke, M. biceps brachii with an EMG activation above the threshold.

**Figure 6:**
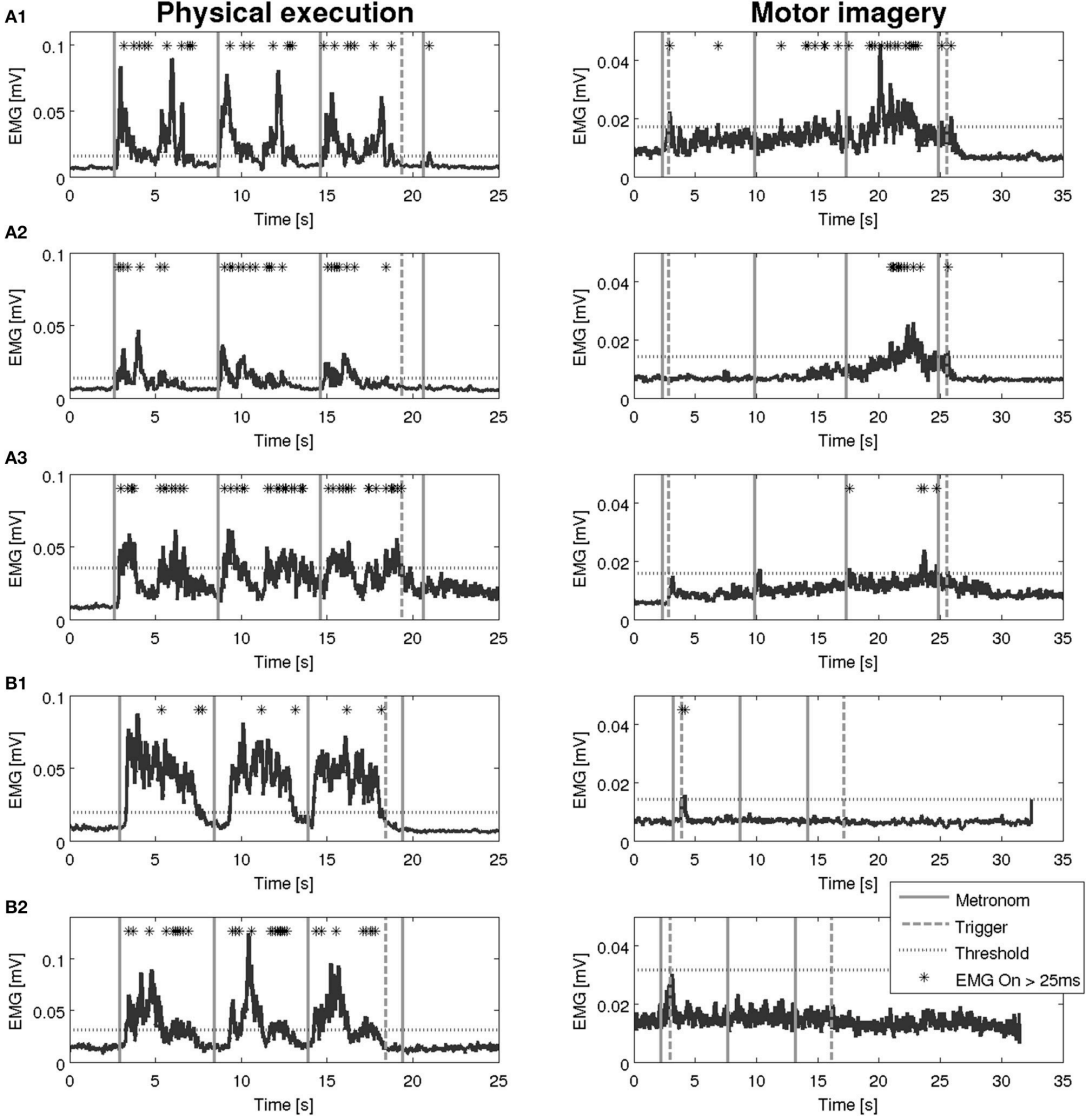
EMG activation during physical execution and MI of two patients with Parkinson's disease. **(A1–A3)** Patient with Parkinson's disease, M. flexor carpi radialis, M. biceps brachii, M. flexor digitorum with an EMG activation above the threshold. **(B1,B2)** Patient with Parkinson's disease, M. deltoideus pars clavicularis with an EMG activation above the threshold, M. biceps brachii with no EMG activation above the threshold.

An overview on the EMG activation during MI and PE is provided in Figure 3. An EMG activation during MI of the hand grasping and arm-lifting task exceeding the threshold was identified in seven out of 22 participants in at least one of the four muscles (two healthy participants, three patients after stroke, and two patients with PD). However, the EMG activation could not be detected in all four muscles and was not present in all three MI trials or in all three repetitions of one trial. Figures 4–6 display the seven participants for each group. Related to Tables 3A,B, we present EMG time series for those patients, where an EMG activation signal pattern could be observed, but the signal remained below the threshold.

#### Detailed EMG activation analysis during MI

The *post-hoc* analysis showed a significantly higher average EMG activation during MI than during the rest condition in two muscles: M. deltoideus pars clavicularis (*z* = −0.743, *p* = 0.001, *r* = 0.414) and M. biceps brachii (*z* = −2.711, *p* = 0.007, *r* = 0.409). No significant differences were found for M. extensor digitorum (*z* = −2.159, *p* = 0.031, *r* = 0.326) and M. flexor carpi radialis (*z* = −2.127, *p* = 0.034, *r* = 0.321).

#### Characteristics of participants, who showed EMG activation

Table 3A present the characteristics of all participants, who did not show an EMG activation during MI and Table 3B presents all participants, who reached an activation during MI.

The seven participants, who showed an EMG activation during MI had a **MMSE** score equal or higher than 28 (max: 30).

- For **MR** six of the seven participants showed a value equal or higher than 60 (max: 64). One patient after stroke scored 51.
- Five of the seven individuals showed a MI/PE ratio within one standard deviation (±SD = 0.28) of all participants (mean = 1.05) in **MC**. The MC score of the other two individuals (1 healthy volunteer, 1 patient with PD) was within two SD above the mean.
- The total **KVIQ** score of the visual and kinaesthetic subscales was 30 (max: 50) or higher in five of the seven participants. The two other participants scored 29 and 28. On the visual subscale six participants had a value higher than 15 (max: 25). On the kinaesthetic subscale four participants scored between 16 and 20, and three scored 15 or below.

### Subgoal analysis

#### Results of MI ability assessment

Please see Tables 2, 3A,B for statistical values on group level and all raw data for each participant):

- For **MR**, differences in the number of correctly identified hand and feet pictures were observed. Healthy volunteers showed a higher correct identification rate than patients after stroke and patients with PD.
- For the **MC ratio**, we observed that more time was needed to imagine than to physically execute the task.
- All participants scored for the visual subscale of the **KVIQ-10** on average 3.3 points higher than the kinaesthetic subscale.

**MI assessments** showed no correlation among each other confirming the different dimensions of MI ability assessed: KVIQ-10 and MR (*r*_*s*_ = 0.130, *p* = 0.564), KVIQ-10 and MC (difference between MI and PE; *r*_*s*_ = −0.146, *p* = 0.518), MR and MC (difference between MI and PE; *r*_*s*_ = −0.142, *p* = 0.529). Furthermore, MI ability scoring of the total KVIQ score was not correlated to the duration of the disease.

#### Relationship between EMG activation, MI ability assessments, and MI quality self-rating

No significant correlation was found between the average EMG activation signal during MI and the scores of MI ability assessments (MR *r*_*s*_ = 0.23, *p* = 0.305, MC *r*_*s*_ = 0.18, *p* = 0.428 and KVIQ-10 *r*_*s*_ = 0.17, *p* = 0.443). Furthermore, no significant correlation was found between the MI quality self-rating after each MI trial and the average EMG activation signal during MI (*r*_*s*_ = 0.01, *p* = 0.984).

## Discussion

The presented study evaluated EMG activation during kinaesthetic MI and PE of a clinically meaningful upper limb hand grasping and arm lifting task, which is an unilaterally executed movement task using open muscle chains. M. deltoideus pars clavicularis and M. biceps brachii showed significantly higher activation during MI than during the rest condition. We observed EMG activations during PE in all participants and an activation above an EMG signal threshold during MI in seven out of 22 participants: two healthy volunteers, three patients after stroke, and two patients with PD. Furthermore, we provided detailed results of all participants' MI ability assessments. However, MI ability assessments showed no correlation among each other and were not correlated with EMG activation.

### Muscle activation during MI

The link between EMG activation and MI was reported with inconsistent findings in the literature. Our results are in line with studies of healthy volunteers (Bakker et al., 1996; Gandevia et al., 1997) and with patients after stroke (Dickstein et al., 2005). In the latter study, the authors mentioned that EMG signal patterns were present during MI in some participants, but could not be recorded systematically in all hemiparetic patients and healthy volunteers. Lacourse et al. did not observe EMG signal patterns during MI of a limb movement (Lacourse et al., 1999). The authors explained their result with the abortion of the task execution by inhibitory processes during MI. The results of the present study support the hypothesis that EMG activation depends on different individual factors and pathways involved in MI. We did not observe overt muscle output during MI indicating a complete motor command inhibition as suggested by Di Renzo at al. based on an investigation with a spinal cord injured patient (Di Rienzo et al., 2014). It is well established that MI suppresses primary motor cortex activation, leading to motor command inhibition. The motor command inhibition may be explained by a strong connection of the supplementary motor area (SMA) and the primary motor cortex, leading to a suppression of primary motor cortex activation during MI (Kasess et al., 2008). However, Page et al. did not report on difficulties to use MI-triggered EMG signals to activate FES in patients after stroke (Page et al., 2015).

An interesting observation of our study is that patients, who showed EMG activation, did so partially with a time lag, i.e., the activation lasted beyond the end trigger and declined 5 to 10 s later.

### Muscle activation and MI ability

McAvinue and Robertson postulated that MI vividness is not an one-dimensional ability (McAvinue and Robertson, 2008). Therefore, our participants' MI ability was assessed with a test battery (MR, MC, KVIQ-10). The majority of our participants reported a vivid and clear image of the hand grasping and arm-lifting task after each MI trial. However, no significant correlations were found between the average EMG signal and any of the MI ability assessments used, nor between disease duration and the total KVIQ score. Our patients after stroke demonstrated a greater variance in the MR scores compared to less variable results in patients with PD and healthy volunteers. That might be explained by the findings from Amesz et al. and Kemlin et al. who reported higher error rates and reaction times for 24 patients 3 weeks after stroke when imaging the affected hand compared to 24 age-matched healthy volunteers (Amesz et al., 2016; Kemlin et al., 2016). Furthermore, Braun et al. found a negative influence of sensitivity loss on the performance of MR scores (Braun et al., 2017). Additionally, the score variance might be explained by the activated brain areas during MR of the body parts: Stroke patients with lower MR scores (<60 points) were mainly affected in the basal ganglia, the frontal cortex and the area of the middle cerebral artery. In our study, patients' lesions were already determined by routine magnet resonance imaging during diagnostic procedures at entry in the acute hospital. Moreover, the variance might be related to the mental slowing after stroke (Malouin et al., 2012). However, the KVIQ scores revealed a similar MI ability performance as reported earlier by Malouin et al. (2007).

Mental chronometry showed comparable MI/PE ratios and a close temporal congruency in patients and healthy volunteers on a moderate to good level of MI ability. These results are in line with findings by Malouin et al. who compared the imagination and execution of stepping movements in patients after stroke and healthy volunteers (Malouin et al., 2008). The generation of accurate forward movement models seemed to be unaffected in both patient groups as postulated by Di Rienzo et al. (2014). Regardless of patients' different affected brain areas and disease duration, the MI ability level evaluated with the MR ratio, the KVIQ-10, and the MI self-rating after the EMG measurements was comparable in patients and healthy volunteers in our study.

### Involved brain areas during MI and included patient groups

Physiological responses during MI were investigated in various brain imaging investigations that included healthy volunteers and patients after stroke. Sharma et al. systematically analysed five functional magnetic resonance imaging (fMRI) studies focusing on imagined and executed hand and arm movements (Sharma et al., 2009). Reviewed studies showed activation in the cortical and subcortical regions that are involved in action planning, execution, and modulation: primary motor cortex, ipsilateral and contra-lateral precentral gyrus and dorsal premotor area, primary somatosensory cortex, and pre-supplementary motor cortex. Furthermore, the positive re-organisational influence of MI on brain connectivity was described in a recent publication by Bajaj et al. (2015). Authors reported an increased inter-regional and network level effective connectivity between the premotor cortex and the primary motor cortex after a 3 week MI intervention focussing on upper limb movement in 10 patients 11 months after stroke. Our patients after stroke showed lesions in the cortical and subcortical regions and were still able to perform MI. However, their MI ability level measured with three MI ability assessments showed varying scores with the lowest scores in the MR assessment and the largest scores in the KVIQ assessment.

### Limitations

One limitation of this study is the small number of participants in each group. Therefore, we avoided group comparisons. It must be emphasised that our study population was a pragmatic convenience sample and were not aged or gender matched, and healthy volunteers were younger than participants in both patient groups. However, seven out of 21 participants showed muscle activation pattern during MI above the threshold of a meaningful and clinically relevant unilateral executed hand grasping and arm lifting task using open muscle chains (2 healthy volunteers, 3 patients post stroke, 2 patients with PD). Our ratio of participants with EMG signal patterns during MI is lower than the one of Dickstein et al. (Dickstein et al., 2005). Dickstein et al. found EMG signal patterns in six out of 15 participants (3 healthy volunteers, 3 patients post stroke) when investigating an alternating, bilaterally executed, closed muscle chain lower limb task involving a limited number of degrees of freedom. However, a type II error cannot be excluded. Future research should investigate a larger number of participants and perform group comparisons. Based on the provided data from Dickstein et al. a sample size calculation with the Gpower software (Cohen's *d* = 0.67, α = 0.05, 1-β = 0.8, 3 groups) resulted in a total of 45 participants for a sufficient group size, with 15 participants per group. Future investigations should furthermore consider factors influencing EMG activation influencing factors, e.g., MI instructions, different tasks to imagine or amount of MI training, and longitudinal changes after an MI training period.

It could be argued that EMG measurements should have been performed during the MI ability assessments too and correlated with the MI ability assessments scores. However, as all patients were inpatients, we intended to keep the measurement/assessment time and burden for the patients as low as possible.

More distinct EMG signal patterns may have been obtained with a task requiring larger muscle activation, for example using a cup with more weight, since studies have shown that EMG activation during MI is proportional to the imagined weight lifted (Guillot et al., 2007). However, for patients after stroke with an upper limb paresis grasping and lifting the provided empty cup was already challenging and heavier objects would not have been feasible.

To familiarise with the MI technique and the hand grasping and arm-lifting task, participants were asked to practice the task two to three times per day between first and second appointment. It would have been interesting to know how many times participants practiced the task mentally between both appointments. Experience from previous MI investigations with patients suggests that the number of mental trials did not exceed the recommended amount of approximately 30 trials (Schuster et al., 2011).

To summarise, seven out of 22 participants (two healthy volunteers, three patients after stroke and two patients with PD) showed an EMG activation during MI of the hand grasping and arm-lifting task in at least one of the target muscles exceeding the threshold. These findings should be confirmed in future investigations, as with the technological and scientific advances MI-induced EMG may eventually become useful in neurorehabilitation. Results of the present study suggest that subliminal EMG activation might be present in seven out of 22 participants (ratio of 1:3.1) in healthy volunteers, patients after stroke and patients with PD. Inconsistent EMG activations may be explained by individual variations. We provided detailed results of all patients' MI ability and could not find a correlation between EMG activation during MI and results of three MI ability assessments.

## Author contributions

MK, BW and CS-A designed the study, helped with the ethics committee application and the interpretation of the statistical analyses and intensively revised the manuscript draft for important intellectual content. Additionally, MK conducted the data. All authors gave final approval of the manuscript to be published and are accountable for all aspects of the work in ensuring that questions related to the accuracy or integrity of any part of the work are appropriately investigated and resolved.

### Conflict of interest statement

The authors declare that the research was conducted in the absence of any commercial or financial relationships that could be construed as a potential conflict of interest.
